# Topics of Public Interest

**Published:** 1906-09

**Authors:** 


### TOPICS OF PUBLIC INTEREST.

### Safeguards to Protect the People from Quack Doctors.

**Exposures of Medical Frauds Do Little Good—General Spread of Education the Best Means for Overcoming Native Credulity.**

By CHAMPE S. ANDREWS,
Counsel for the Medical Society of the County of New York.

(*New York Tribune, Sunday, August 5, 1906.*)

"ONE would think that with the spread of scientific knowledge fewer people would be taken in by quack doctors, yet it has apparently made little difference with the prosperity of these frauds," continued Mr. Andrews. "The man or woman who would laugh at the 'ramcats' and 'well-my-gristle,' with which 'Dr.' Thompson victimized New Englanders a century ago, is as likely today to buy a piece of red flannel denominated a liver pad or to invest in a pair of 'magic boots.' And the field is so lucrative that our detectives are always busy in the campaign against these medical crooks.

The average American does not think very far. He reads in his favorite paper about the discovery of radium or the *X*-ray and some of the wonderful properties of these curious *substances* and radiations. It has nothing to do with his business, so he does not think further about the discoveries. His curiosity has been satisfied, and that is enough.

It is different with the quack. Every time a new scientific discovery is reported in the press thousands of quacks are made. Think of the radium and electrical frauds which have been successfully perpetrated. The quack is keen witted, as he must be in order to succeed. He knows how to make what knowledge he has count. It is business with him. In his shrewdness he has learned to adapt his methods to the times, and that the way to succeed is to imitate true scientific methods. He must at least be so plausible that he will overmatch the half knowledge of his victims. The very fact that science is advancing is a part of his stock in trade, for the general knowledge of this fact fits his prey to accept his statements about the wonderful new remedies based on the latest discoveries in science.

The well known medical fraud who died the other day after serving a term for swindling a Mount Vernon man is an illustration of the evolution of a reputable physician into a swindler, and how the great scientific discoveries may be turned into the service of the quack. After passing through an American college with honors, this future quack went to Europe and made special studies in medicine. When he came back he became a member of the County Medical Society. His ability was such that he sometimes addressed the society on medical topics. He was considered so learned that he was decorated by a foreign society.

The legitimate practice of the medical profession, however, did not promise to give him wealth speedily enough. In course of time he was expelled from the County Medical Society for questionable practices. He accumulated several fortunes, and was once president of the Road Drivers' Association. On one occasion he was toastmaster of a banquet at which Admiral Dewey was the chief guest.

USED KNOWLEDGE TO SWINDLE.

He used his skill and knowledge to dupe men and women. For instance, he invented a machine which he called a dynostat. Note the scientific character of the name. This he described in the circular which he issued as 'A New Electrical Force that Gathers and Intensifies the Strength of Drugs and DRIVES THEM INTO ANY ORGAN.' Here is a copy of the circular. Here are some of its statements:

"Science in the course of its marvelous advancement has placed within our reach a means of using both drugs and electricity, each in its best and strengthens the other and the one helps and strengthens the other, with the result that previously hopeless cases *get* well and *stay* well. By means of the dynostat we are enabled to reach easily and medicate *perfectly* and *harmlessly* organs of the body without the use of stomach drugging, and to reach and cure diseases that neither electricity, galvanism or drugs could heretofore possibly cure. It is a marvel of electrical skill. Its use in conjunction with the proper remedies, is one of the most startling discoveries in medical science at the present day. It is as wonderful, from a medical point of view, as the *X*-ray was from a scientific point of view."

There is a plausible display of scientific knowledge for you! Note how he carried this argument to a logical conclusion. 'By means of the ray,' the circular continues, 'light is made to penetrate bodies heretofore impenetrable by light.' Observe the black faced type of the circular in which what follows is printed. If you have had your doubts of the greatness of the discovery before now have the proof, that the type seems to say. The circular says:

"By means of the dynostat certain drugs may be driven clear through a person's body. For instance, a solution of methyline blue, painted upon the abdomen of a person will, in five minutes, under the influence of the dynostat, disappear entirely, and by a reversal of the current, the same drug may be made to appear on a patient's back. Thus, it will be seen, the dynostat has the power not only to drive a drug into the system, but to draw it out as well. The dynostat can in many cases (by reversal of current) draw out disease and disease germs, the same as it does in the case of coloring matter or drugs.

An indubitable proof of the penetrating power of drugs used in conjunction with the dynostat is the fact that if a patient with a cough and expectoration is operated upon by it, in five or ten minutes the coloring matter that has passed through the body and into the lungs will appear in the expectorated sputum."

That fraud knew that there was nothing like showing a prospective dupe that the instrument would do something.

'Seeing is believing,' was his argument.

CLIMAX OF CAREER IN JAIL.

The climax of his career came a year ago, in January, when he pleaded guilty to a complaint brought by a Mount Vernon man whom he had swindled out of $10,000, and began the service of a sentence of imprisonment of four months. The game he worked on the Mount Vernon man was this:

A couple of years ago, perhaps, the West Chester man received a visit from a man supposed to be a life insurance agent. The latter persuaded him to apply for insurance. He was examined, but was not accepted, according to the insurance agent, who was in league with the inventor of the dynostat, because of his physical condition.

'I can introduce you to a doctor, however, who can fix you up so that you can get in,' said the agent. 'He's got a wonderful invention that can tell you just what the trouble is.' The Mount Vernon man accordingly met the doctor with the dynostat. The doctor persuaded him to buy one of the machines, and he gave him small doses of strychnine. Following the course of strychnine the Mount Vernon man was dosed with morphine. This broke him up. Then the dynostat man said he would introduce him to a Doctor Hale, an English specialist who happened to be in this country. 'Perhaps' he said, 'Dr. Hale will undertake to diagnose your case, although he is a very busy man.'

The Mount Vernon man went to see the celebrated specialist. On the first visit he waited in the office a long time, and was obliged to go away without seeing the alleged physician. They were playing him and he was hanging onto the hook well. It was not until the third visit that he was admitted to the presence of the great man. As he entered he observed lying on the desk in a conspicuous place a long, white cardboard box. It was open. On a bed of cotton within it was a vial filled to within an inch of the top with a greenish yellow fluid. As you see" (the lawyer held up a hermetically sealed bottle, filled with a liquid suggestive of a beautiful engine oil), "it was hermetically sealed when I got it," he continued.

The major part of the diagnosis," he went on, "consisted of a series of questions intended to disclose the sum the patient could pay for a cure. Then the learned Englishman announced that the patient had Bright's disease. There was only one thing, he said, that would cure the disease. That was radium. As he said this he took up the bottle of greenish-yellow liquid from its soft bed and held it up before the light.

'Do you see the shining particles floating in that liquid?' he said. 'They are particles of radium.' The great man fingered the bottle lovingly as he added: 'That bottle contains radium valued at hundreds of thousands of dollars. I could let you have a little of this if you had the money. In order to help you to a cure I will contribute an amount of radium equal to that which you can afford to buy.'

The man did not know what to do. He believed that he was suffering from a disease which could be cured only by the use of the expensive compound. He thought of the few thousands of dollars he had laid up against a rainy day and for the comfort of his son. The argument, What is your money to be compared with your life? finally brought him to terms, and it was agreed that he should have a small bottle, about the size of your little finger, filled with the precious, life-giving fluid, for $10,000, his entire savings. The money was paid and he began to take the medicine three drops in a wine glass of water three times a day. The victim came to the conclusion too late that he was a dupe, and he had the man who invented the dynostat arrested. I have shown the bottle to chemists, and they say that it contains a mixture of copper.

CHARACTER OF VICTIMS

The people who promise the most as victims are those who are either hypochondriacs or who are suffering from some disease which has wasted away their powers of mental resistance to such an extent that their minds are openly receptive to the arguments of the charlatan and they are ready to build up illusive hopes upon his marvelous claims and extravagant promises. The hypochondriac is so fruitful a field that in the operations of the quack there is a place for the 'hypo creator,' as those are styled who devote their energies to creating in people a belief that they are sick. The real sufferers who may be depended upon to furnish patients for the quack include the hopeful consumptive and the sufferer from locomotor ataxia. Many of these are ready to grasp at any straw as they draw near the brink of the fall. In fact, the medical mountebank is no better than a straw in his ability to save those who are within sight of the edge—or to heal those who are not, for that matter.

For the benefit of the consumptive, the quack has followed his usual course and drawn upon science for his bait. Some time ago advertisements appeared of an *X*-light which had been designed for examination of the lungs in order to determine whether a person had consumption or not. By means of the invention the physician was supposed to be able to see into the lungs and determine if there was any diseased tissue there. In the advertisement attention was drawn to the fact that the earlier the presence of the disease could be determined the easier it could be cured. No matter how slight the lesion, it could be discovered by the *X*-light.

After using his *X*-light the quack advertiser would bring forth a picture of a pair of diseased lungs, which he said was the picture he had just made of the patient's breathing apparatus. This was a fine 'hypo creator.' The patient could not know that the solemnly given diagnosis of the diseased condition of the lungs was only an invitation to take his vibratory treatment, ultra-violet rays, electricity, etc.

Electricity has been worked hard by the quack. Large sums of money have been made in the sales of batteries, magnetic belts and shoes with zinc and copper insoles. It has been called 'the stream of life,' a 'mysterious force' and other fanciful names by innumerable swindlers. The dynostat was based upon it.

Two or three months ago the papers contained accounts of a man who had made 'magic boots' for those with deformed limbs. He had been arrested on the charge of grand larceny by false pretense, etc. For some of these boots the maker is said to have secured $5,000 a pair. He declared that they possessed a mysterious power which would cure such diseases as locomotor ataxia, paralysis, rheumatism and neuritis. When asked what the 'mysterious power' was he would talk darkly about their being 'charged' so that when worn they set up a magnetic influence that caused a current of nerve force to course through the body. The shoes could be 'recharged' by him—at a 'charge' of $25. Their real and only value lay in the fact that, having been made to fit plaster casts of the feet of the prospective wearers, they fitted their feet exactly and bore the weight comfortably without regard to the position in which the feet received it.

In his establishment he had a room which he called the 'Chamber of Miseries.' It reminded one of a shrine, for its walls were hung with braces of all kinds, which he would say to the visitor had been taken from cured patients. Among the braces was one of iron which he declared had been worn by Bishop Potter, but which he had replaced with a pair of his 'mechano-physiological boots.'

Professor F. B. Crocker, head of the electrical department of Columbia University, testified at the preliminary hearing before Magistrate Poole that the shoes could hold no electricity and were like ordinary shoes. The minimum price of this footgear was $200 a pair, and, as the evidence showed, there had been not a few pairs sold at prices higher than this.

WOMEN WATCH FRAUDS.

To detect and convict these medical crooks and illegal practitioners the County Medical Society maintains an experienced detective force and a remarkable card index system for keeping tabs on this class of criminals. Detective work involves temptation, and especially so when it is carried on against wealthy swindlers who are able to corrupt the emissaries of justice. Every one in charge of a force of investigators realizes the difficulty of keeping them honest and efficient. We had had more or less trouble with our agents, or detectives, and four had been discharged for suspected collusion with the men they had been sent to watch. We had an ex-sergeant of cavalry as chief detective. He was a very able man, and is now in the Police Department. In some delicate cases he had his wife assist him in procuring evidence, with such success that several of her friends —stenographers, governesses, telephone operators and trained nurses—were pressed into service for occasional cases. Eventually women practically excluded men altogether from our force of detectives, and since then not a case of dishonesty has come up.

The society has ten women agents, and they have shown themselves ideal detectives, endowed as they are by nature with all the arts of dissimulation. Their wits match those of the wiliest charlatans on the lookout for legal retribution. What man could extract evidence with a winning smile or tell a long fiction with entire plausibiltity? It might seem that men have the advantage in disguising themselves with more or less whiskers, but the woman detective, by a change of costume and voice, dropping her hair from pompadour so as to conceal brows and ears, can transform her appearance even more radically. Again, a majoriity of medical sharks prey on women, which makes it easier, as well as poetically just, for the feminine sleuth to compass their downfall.

The only fault of women detectives is that of indiscretion," Mr. Andrews declared, "and this has led to a few compulsory resignations. When an agent becomes too familiar with opposing counsel or a prisoner, acts in court in such a skittish way as to discredit the serious work of the society, gives interviews to newspapers without authority or tells her husband about an impending case—which has happened and been discovered—her usefulness is at an end. It may appear a harsh rule not to confide in husbands, but when one considers that great interests are at stake and fakers have thousands of dollars to spend in getting information, its wisdom becomes obvious."

'Every flea has another flea to bite him,' wrote the poet, and the County Medical Society frankly watches one set of detectives with another. This is the time honored system of the Petersburgh Third Section and the Washington Secret Service. Two agents are often sent to the same place at different times without the knowledge of each, and their reports are compared. It frequently surprises an agent in court to find a sister investigator giving testimony. But the principal object of double espionage is to keep the emissaries of quacks from getting on the staff and checkmate the efforts of four or five quacks' sleuths who dog the steps of the society officers for the purpose of getting information. The charlatans, realizing the convenience of having news direct from the enemy, have sent stylishly dressed, handsome women to the society's offices as applicants for detective jobs. These women have been traced back to the fakers' headquarters.

### MARRIED WOMEN PREFERRED.

The preferred agent is a married woman of good repute, but without previous experience, who can be trained to the work in hand. She is put on probation for a period, and learns the elements of the law of evidence, the difference between hearsay, or loose conjecture, and the hard facts needed to secure conviction. She goes out with an old agent and writes reports to test her memory and seeing talent. After a while she is used as a corroborative witness, and in four to six months' time becomes competent to handle independent investigation. Once employed, the agent never comes to the society office, but is communicated with at her home. This is a measure of precaution. The daily reports are filled out according to this form:

Statement of............... Residing at...............

On the .....day of ............., at...o'clock, I visited

..................., whom I found located in ............., at No. ..............Street. A sign on the.................. read as follows:............ On entering I was met by (Here state, in order mentioned, what defendant said at introduction, what symptoms you gave, how he examined you, what his diagnosis and treatment, and whether you received anything in writing from suspect).

The suspect's card read as follows, and is hereto attached ................ The suspect's advertisement and literature is hereto attached and initialled by me. The medicine given by the suspect consisted of........, and has been initialled by me. I paid the suspect $.... in the form of............

During my interview and treatment there was present with me........ No other person was present except............. Remarks.............

(Signed, with date).

The agent is supposed to write her report immediately after visiting the quack's office, whether in a convenient hallway, at a drug store counter or a hotel table, so as to insure accuracy of all conversations. No badge or weapon is ever carried by the agent, and in most cases she does not make her identity known until the confrontation in court. There are assuredly unpleasant and a few positively dangerous moments that try the nerves of the investigator and make her wish she was not alone in the power of a scoundrel who might contemplate any crime to escape prison. These moments are especially liable to occur when a trap is set for the charlatan and the authorities break in at a critical point upon a signal. It happened once that a helpless agent had to disclose herself when a signal went wrong and the officers were delayed in entering.

'Mary, put those instruments away,' ordered the malefactor to a servant.

'You'll do nothing of the kind,' said the pseudo patient. 'I am an agent of the Medical Society, and I tell you to leave everything alone under penalty of the law.'

The quack and his servant gave the agent a murderous look, but did not venture to translate their feelings into action.

Detective Sergeant Andrew Ferretti is detailed by Police Commissioner Bingham to head the society's detective force and make all arrests. He and the one other man on the staff also attend to all 'trailing.' Speaking four languages, Detective Ferretti is especially skilled in Italian dialects, the use of which assumes importance in many midwife cases. The first thing ascertained in such a case is which dialect out of a dozen or so is spoken by the suspected person. If Ferretti doesn't know the exact dialect, some member of the staff does and goes out to win the person's confidence and get evidence.

NEW TRAP EFFECTIVE.

The officers of the society pride themselves on the economy, neatness and dispatch of a newly devised scheme of trapping a certain dangerous kind of practitioner. According to the old way they had to hire apartments and to pay for an expensive 'plant,' witnesses and detective being concealed in a closet or under a bed. Now, the hapless grafter is made to supply the 'plant' in his own house. There is no expense for rent. Any marked money is returned immediately after the arrest.

'It's tough to think of the same $20 bill catchin' half a dozen of us,' remarked one crooked practitioner after his arrest.

One agent, in the new method, arranges to have an illegal operation performed at 11 o'clock in the morning. At 10:45 o'clock Agent No. 2 enters the office and ostensibly waits to see the doctor. A few minutes after 11 Detective Sergeant Ferretti and John S. Cooper, a lawyer, arrive in the street and either wait in a hallway or loiter in a store opposite. There is an elaborate code of signals. If anything went wrong with No. 1, she could pass out of the office and, without saying a word, let the second agent know that she must complete the job. But if all goes well No. 1 gives a signal at the critical moment, perhaps by a natural yet piercing scream or a handkerchief shown at a window; whereupon the pseudo patient No.2 opens the outside door to admit detective and lawyer. And the malefactor is caught redhanded. Nothing is lacking to the artistic plot of justice that resembles the confederated system of crooks—a stool, a 'pal,' a police officer with power of arrest and a lawyer to manage everything according to legal etiquette.

As a detail of economy it has been discovered that a quack whose regular fee is $10 will accept $2 or even 50 cents and a promise. The agent is generally as liberal of her promise to pay as he is of his engagements to cure. But the payment of a fee is not a necessary feature in legal evidence.

About one in every four cases investigated is rejected by the society. Victims and revengeful persons bring in many complaints, and cases are followed up from newspaper advertisements of charlatans. Few victims are willing to tell their experiences on the witness stand which makes it necessary for the detective to collect nearly all the evidence. A case once taken up is never dropped. From three hours to one year is required for an arrest. The three hour record was made one morning by Detective Ferretti and Mr. Cooper, on receipt of a complaint forwarded by Police Commissioner Bingham. Within the time named the quack had been investigated, had confessed and been locked up. A quick job is usually engineered by sending in three or four women agents in succession. By the time the last one is out of the quack's office enough evidence to convict is in the hands of the lawyer. Sometimes an agent is instructed to act in a suspicious manner, or make the quack angry, so that he may betray himself in some way. Having done her part as irritator, she leaves the place for good, and a smooth tongued agent completes the work.

It takes three or four months to get up a case against a diploma mill, where the proprietor is too wary to sell his diploma for immediate cash. In the case of a Broadway School of Chiropody an agent was enrolled as a scholar, took the regular three months' course, obtained a diploma and thus had evidence against the institution. Osteopathy medicos have been lured to the homes of agents who became suddenly ill. Sometimes a bluff is resorted to instead of an arrest when the evidence does not seem sufficient. For example, a woman physician was put through an informal third degree in her office. She broke down, wept, confessed and promised to leave town.

### MANY CLASSES OF CROOKS.

The charlatans operating in New York are classified as consumption sharks, advertising specialists for men only, oculists, nature and water cure doctors, midwives, osteopaths, somatopaths, vitapaths and other "paths" too numerous to mention. The man who is known to have money is the especial prey of these quacks. A short time ago, an illiterate but shrewd Irishman, of this city, who had accumulated a little fortune by his investments in real estate and the construction of small apartment houses, died from locomotor ataxia. For some months before it had been almost impossible for him to speak. To the average quack he looked like a good subject. One day a stranger called at his home and told him about some wonderful underwear which, if worn by one afflicted with locomotor ataxia, would cure him. The price, the stranger said, was $250 a suit, but in view of what it would accomplish, that was a moderate figure.

The habits of a lifetime clung to the old Irishman, and he declined to pay 'sight unseen.' The stranger asked the privilege of bringing an old man who had been cured by wearing the underwear to prove all that was claimed for it.

The second floor of the Irishman's home was undivided, the entire floor being used as a drawing room. There was not a great deal of furniture in the room, and it was an excellent place for the display which the stranger had arranged in order to convince the well-to-do Irishman. On the day appointed the stranger came with an elderly appearing man. The latter gave an exhibition in the long drawing room that would have done an acrobat credit. The old Irishman was still suspicious of the truth of the assertions of the seller of the underwear, for there was something about the appearance and the actions of the gray-bearded gymnast that suggested that he was a professional of possibly twenty-five years made up for the part he assumed. He did not buy any of the high priced underwear.

The laws against medical fakers are held to be much too lenient. Practising medicine without a license is merely a statutory misdemeanor, and the penalty is $250 fine or six months in prison for the first offence, with double the amount of fine and imprisonment, either or both, for the second offence. It is fortunate that a charge of grand larceny can be made against those who ruin a patient's health and then charge several hundred or a thousand dollars for the service. No penalty would seem too great for a charlatan who deliberately creates running sores on the body of a healthy man, then tortures his mind and robs his purse under the delusion that he is suffering from an incurable disease.

### MANY FRAUDS LAUGHABLE.

Some of the ways in which mankind is willing to be duped are almost unbelievable, they seem so ridiculous. Disease is not the only source of revenue of the fraud. There are those who are suffocating from an oversupply of sentimentality, and who believe in supernatural powers. For such a 'Dr.' White, of Baltimore, who was tried recently in the United States District Court in that city on the charge of using the mails to defraud and is now serving a three years' sentence, compounded love charms and invented the 'ancient Egyptian breastplate,' said to contain charms and prayers. The latter was said by the 'doctor' to be charged with 'magic solar fluid.' The breastplates were two inches square. They were filled with colored fullers' earth. Printed prayers were sewn into them. This plate would do many things for the wearer, according to a circular which was read in the court It would right love affairs which were not progressing favorably. It would turn the tide in one's business. It would give health to the sick. In the words of the circular:

"It will draw to you great forces and enable you to gain many of your greatest desires. It will give you good luck and guide you to success. It will lead you to the greatest secrets. It will cause you to learn all secrets. It gives you magical power. It will cause you to gain friends and also enable you to gain money from all parts of the country.

Your purse will be fuller.
Your life will be brighter.
Your home more happy.
Your desires will be granted.
Bad will turn to good.

According to the testimony, White also made mirrors for the use of mediums by which they could see spirits. One woman said she paid 'Dr.' White $700 for a pair of them. A carpenter demonstrated the profits in the quackery business by testifying that he made the mirrors, and the cost was $3.50 each.

The spell to create love was styled the 'Adam and Eve' charm. Adam and Eve were represented by roots declared to be obtained from Africa—Adam was one root and Eve another. The roots were to be placed in running water and the words 'Whom God hath joined together let no man put asunder' were to be recited.

MANY PERSONS SOUGHT CURES.

At the trial which resulted in the conviction of the 'doctor' more than 200 persons who had been duped in one way or another were present to testify. They came from widely separated parts of the country. There were cripples who had thought to secure help from this wonderful man; there were clergymen who had sought cures; there were young women, one an attractive Brooklyn girl, who was manager for a tiling business and had had a difference with a 'gentleman friend,' who had sought help in untangling their knotty love affairs; there were members of families whose homes had been broken up by the coming of the 'doctor's' correspondence course in black and white art. The Brooklyn girl was more fortunate than some of her fellows, for the $5 which she had sent for the 'Adam and Eve' charm was returned, as the 'doctor' said he was out of the roots which comprised it. One woman who had taken the course in the magic arts, with the expectation of discovering a pot of gold in the earth just outside the house and of becoming a spirit medium, became so suspicious of the presence of spirits that she practically became insane, and was so near death that a priest administered the last sacrament. He discovered the book sent to the woman by 'Dr.' White which had caused all the trouble, and after he had burned it the woman began to recover. She left her home and had not returned at the time of the trial. Although separated, the woman, her husband, their son and the priest were all at the trial as witnesses.

It is estimated that between 15,000 and 20,000 people in different parts of the country had become students in his correspondence school for instruction in 'spiritualism, hypnotism and black and white arts.'

There was a great hubbub in the Yorkville Police Court in the latter part of March. A score or more of the East Side women had gathered there to testify against Julius Benjamin. He was charged with practising medicine without a license. It was asserted by one of the witnesses against him that he had a silver handled broom which he used in brushing away the sins of those who came to him for advice, and seemed to be so laden with guilt that nothing could be done for them until the cobwebs of error had been brushed away.

There are hundreds of people in New York who feel that they have been benefited by the 'prayer strips' which one East Side 'healer' has given them. These 'strips' are pieces of paper on which are written the prayers which the 'healer' offers up for the health of her patients before an altar in her room. What the prayers are, are unknown to those who are depending upon them for health, for they are in characters with which they are unfamiliar.

The man with thousands of dollars and the poor with only 15 cents to pay for a 'prayer strip' alike are vulnerable to the arts of the quack and the fraud. Altering Puck's comment slightly: 'Lord, what fools some mortals be.' "

---

The Administration of Drugs for the Concurrent Retardation and Stimulation of Systemic Oxidation.—Bernard Oettinger believes that a methemoglobinemia of nontoxic proportions may exist, and hence that this blood condition must not invariably be regarded as deleterious to conservative metabolism. He shows that good comes to a patient from slight methemoglobinemia, effecting an increased oxidation of a selective character. This is an oxidation through methemoglobin of intermediate products of metabolism, which in diseased conditions accumulate in abnormal amounts in the blood, but which, at the same time, is an oxidation without effect on the normal tissues. Therapy makes use of this blood change to aid in conservation of nutrition. As examples of the drugs used for this purpose the writer cites, among others, amyl nitrite for angina pectoris, and pyridin inhalations in asthmatic attacks. In connection with the foregoing hypothesis the writer advances a second to explain rationally the concomitant use of another class of drugs, whose relation to systemic oxidation is opposed to those already mentioned. Quinine, arsenic, phosphorus, iron, and strychnine are used in both acute and chronic diseases. The first three are given individually, under widely different indications. Their concomitant use, the writer believes, is due to the fact that they have in common the property of retarding tissue oxidation. In disease, regressive metabolism is always increased. Against such increased catabolic tissue oxidation the use of a drug which retards oxidation must be valuable. Thus is explained the simultaneous use of drugs to effect concurrent retardation and stimulation of oxidation.—*Medical Record, July* 28, 1906.

